# Increased circulatory levels of fractalkine (CX3CL1) are associated with inflammatory chemokines and cytokines in individuals with type-2 diabetes

**DOI:** 10.1186/s40200-017-0297-3

**Published:** 2017-04-04

**Authors:** Sardar Sindhu, Nadeem Akhter, Hossein Arefanian, Areej Abu Al-Roub, Shamsha Ali, Ajit Wilson, Asma Al-Hubail, Shaima Al-Beloushi, Saad Al-Zanki, Rasheed Ahmad

**Affiliations:** 1 Immunology Unit, P.O. Box 1180, Dasman, 15462 Kuwait; 2 Islet Biology Unit, P.O. Box 1180, Dasman, 15462 Kuwait; 3 Clinical Laboratory, P.O. Box 1180, Dasman, 15462 Kuwait; 4 Animal & Zebrafish Core Facility, Dasman Diabetes Institute (DDI), P.O. Box 1180, Dasman, 15462 Kuwait

**Keywords:** Fractalkine, CX3CL1, Chemokines, Cytokines, Inflammation, Obesity, Type-2 diabetes

## Abstract

**Background:**

Fractalkine (CX3CL1) is involved in the development of numerous inflammatory conditions including metabolic diseases. However, changes in the circulatory fractalkine levels in type-2 diabetes (T2D) and their relationship with inflammatory chemokines/cytokines remain unclear. The aim of the study was to determine the T2D-associated modulations in plasma fractalkine levels and investigate their relationship with circulatory chemokines/cytokines.

**Methods:**

A total of 47 plasma samples were collected from 23 T2D and 24 non-diabetic individuals selected over a wide range of body mass index (BMI). Clinical metabolic parameters were determined using standard commercial kits. Fractalkine and chemokines/cytokines were measured using Luminex X-MAP® technology. C-reactive protein (CRP) was measured by ELISA. The data were compared using unpaired *t*-test and the dependence between two variables was assessed by Pearson’s correlation coefficient (r).

**Results:**

Plasma fractalkine levels were significantly higher (P = 0.005) in T2D patients (166 ± 14.22 pg/ml) as compared with non-diabetics (118 ± 8.90 pg/ml). In T2D patients, plasma fractalkine levels correlated positively (P ≤ 0.05) with inflammatory chemokines/cytokines including CCL3 (r = 0.52), CCL4 (r = 0.85), CCL11 (r = 0.51), CXCL1 (r = 0.67), G-CSF (r = 0.91), IFN-α2 (r = 0.97), IL-17A (r = 0.79), IL-1β (r = 0.97), IL-12P70 (r = 0.90), TNF-α (r = 0.58), and IL-6 (r = 0.60). In non-diabetic individuals, fractalkine levels correlated (P ≤ 0.05) with those of CCL4 (r = 0.49), IL-1β (r = 0.73), IL-12P70 (r = 0.41), and TNF-α (r = 0.50). Notably, plasma fractalkine levels in T2D patients associated with systemic inflammation (CRP) (r = 0.65, P = 0.02).

**Conclusions:**

The altered plasma fractalkine levels associate differentially with inflammatory chemokines/cytokines in T2D patients which may have implications for T2D immunopathogenesis.

**Electronic supplementary material:**

The online version of this article (doi:10.1186/s40200-017-0297-3) contains supplementary material, which is available to authorized users.

## Background

The chronic low-grade systemic inflammation associated with obesity called as metabolic inflammation is a contributory factor to the induction of insulin resistance and development of type-2 diabetes (T2D) and also leads to other clinical complications of metabolic syndrome [1, 2]. Altered circulatory levels of various chemokines, proinflammatory cytokines, and adipokines have been widely reported both in obesity and T2D [3–5]. The proinflammatory cytokines and chemokines are involved in the pathogenesis of a wide variety of inflammatory disorders. Macrophage inflammatory proteins (MIP) belong to CC-motif chemokines and the two isoforms called MIP-1α (CCL3) and MIP-1β (CCL4) are induced by exposure of macrophages to endotoxins and the elevated expression of these inflammatory proteins was observed in primary monocytes obtained from T2D patients [6]. Similarly, the increased circulatory levels of CXC-motif chemokines were reported in patients with type-1 diabetes [7, 8]. The elevated circulatory levels of CXCL1 and CXCL5 in T2D patients [9] and the increased CCL11 (Eotaxin) protein in serum and mRNA in visceral adipose tissue of obese mice and humans have been reported [10]; and these chemokines may affect the recruitment of eosinophils, basophils, neutrophils, and monocytes at the site(s) of inflammation. These immune regulatory cells synthesize and release proinflammatory cytokines such as IL-1, IL-6, and TNF-α which are regarded as the key factors involved in metabolic dysregulation including glucose and lipid disorders and insulin resistance in T2D [11–13]. The T-helper (Th)-17 cells express proinflammatory cytokine IL-17 which regulates adipogenesis, glucose homeostasis and obesity [14]; and it has been also implicated in the pathogenesis of several inflammatory diseases including rheumatoid arthritis, psoriasis, systemic sclerosis, and T2D [15–18].

Fractalkine, also known as CX3CL1, is a chemokine with chemotactic activity for monocytes, T cells, and NK cells, all of which play important roles in the development of numerous inflammatory conditions including arthritis, atherosclerosis, insulin resistance and T2D [19–21]. However, it is not clear whether the plasma fractalkine levels are modulated in T2D, and if so, how do these changes relate with the inflammatory chemokines and cytokines in diabetic patients. Therefore, the aim of the study was to determine the circulatory fractalkine levels in T2D and non-diabetic individuals and to also find the relationship between plasma fractalkine and inflammatory chemokines/cytokines in these subjects. Herein, we present the data showing significantly higher plasma fractalkine levels in T2D patients as compared with non-diabetic controls. Furthermore, these changes correlated positively with signature inflammatory chemokines and cytokines in the circulation.

## Methods

### Study population

A total of 47 individuals comprising of 23 diabetic and 24 non-diabetic adults were recruited in the study by attending physician at the outpatient clinics of Dasman Diabetes Institute (DDI). The clinico-demographic characteristics of our study cohort are summarized in Table 1. Those younger than 18 years or older than 60 years were excluded from the study. The individuals suffering from type-1 diabetes, or with serious heart, liver, kidney, and lung diseases or with immunological disorders, malignancy or pregnancy were also excluded. All participants gave written informed consent prior to enrolment and the study was approved by the DDI research ethics committee. The T2D was diagnosed by recruiting physician based on results of fasting blood glucose (FBG), oral glucose tolerance test (OGTT), and glycated hemoglobin (HbA1c) test. The FBG levels of ≥126 mg/dL (≥7 mmol/L), 2 h-OGTT values of >200 mg/dL (11.1 mmol/L), and/or HbA1C levels of ≥6.5% on two separate tests were diagnosed as T2D positive. Anthropometric and physical measurements included body weight, height, waist and hip measurements. Height and weight were measured with barefoot participants wearing light indoor clothing using calibrated portable electronic weighing scales and portable inflexible height measuring bars. The waist circumference at the highest point of the iliac crest and the mid-axillary line was measured using constant tension tape at the end of a normal expiration with arms relaxed at sides. The waist-to-hip ratios were calculated and the whole-body composition including body fat percentage, soft lean mass and total body water were measured by using IOI 353 Body Composition Analyzer (Jawon Medical, South Korea). The body mass index (BMI) was calculated using standard formula as follows. BMI = body weight (kg)/height (m^2^). Regarding clinical laboratory measurements, peripheral blood was collected by designated phlebotomist through venipuncture from the overnight (minimum 10 h) fasted individuals and samples were analyzed for FBG, HbA1c, and lipid profile. Glucose and lipid profiles were measured using Siemens dimension RXL chemistry analyzer (Diamond Diagnostics, Holliston, MA, USA) and HbA1c was measured by using Variant device (BioRad, Hercules, CA, USA). All assays were carried out following instructions from the manufacturers.

**Table 1:** Patients’ demographic characteristics and clinical data

Parameter	Diabetic			Non-diabetic		
	Lean	Overweight	Obese	Lean	Overweight	Obese
Total number (N)	8	8	7	8	8	8
Male (N)	4	5	6	2	3	1
Female (N)	4	3	1	6	5	7
Age (Yrs.)	48.5 ± 3.7	48.0 ± 2.8	51.3 ± 5.9	38.5 ± 4.4	42.6 ± 3.7	40.5 ± 4.5
Body mass index (Kg/m^2^)	23.4 ± 0.6	27.3 ± 0.5	33.1 ± 0.7	22.6 ± 0.7	27.4 ± 0.7	35.9 ± 1.6
Body fat (%)	30.0 ± 3.1	31.4 ± 2.7	34.9 ± 1.4	29.7 ± 1.8	35.1 ± 1.3	41.9 ± 1.4
Waist circumference (Cm)	76.4 ± 3.0	90.0 ± 5.5	113.4 ± 1.6	78.2 ± 3.2	94.5 ± 3.4	110.1 ± 2.3
Waist to hip ratio	0.82 ± 0.05	0.90 ± 0.03	0.96 ± 0.04	0.81 ± 0.03	0.95 ± 0.01	0.97 ± 0.02
Fasting plasma glucose (mmol/L)	8.0 ± 1.1	7.4 ± 0.7	9.2 ± 1.1	4.9 ± 0.1	5.3 ± 0.2	5.7 ± 0.3
Glycated hemoglobin (HbA1c) (%)	7.5 ± 0.8	6.8 ± 0.5	9.2 ± 0.7	5.4 ± 0.3	5.7 ± 0.1	5.4 ± 0.2
Total cholesterol (mmol/L)	5.6 ± 0.4	5.0 ± 0.5	5.6 ± 0.7	5.1 ± 0.3	5.5 ± 0.3	5.0 ± 0.3
High-density lipoprotein (mmol/L)	1.5 ± 0.4	1.2 ± 0.1	1.1 ± 0.2	1.5 ± 0.2	1.4 ± 0.1	1.4 ± 0.1
Low-density lipoprotein (mmol/L)	3.5 ± 0.2	3.2 ± 0.5	3.4 ± 0.6	3.1 ± 0.2	3.5 ± 0.3	3.0 ± 0.2
Triglycerides (mmol/L)	1.5 ± 0.3	1.4 ± 0.3	2.1 ± 0.8	0.7 ± 0.1	1.2 ± 0.2	1.2 ± 0.2

### Determination of plasma chemokines/cytokines

A total of 41 chemokines and cytokines were measured using panel MILLIPLEX MAP Human Cytokine/Chemokine Magnetic Bead Panel-Premixed 41 Plex-Immunology Multiplex Assay (Milliplex map kit, HCYTMAG-60 K-PX41; Millipore, USA) following the manufacturer’s instructions. Data from the reactions were acquired using Luminex, Milliplex analyzer while a digital processor managed the data output and Milliplex analyst software was used to determine the mean fluorescence intensity (MFI) and analyte concentration (pg/mL).

### Statistical analysis

The data obtained were expressed as mean ± SEM values and group means were compared using unpaired *t*-test. The linear dependence between two variables was assessed by Pearson’s correlation coefficient (r). GraphPad Prism software (Version 6.05; San Diego, CA, USA) was used for statistical analysis as well as for graphical representation of the data. All P-values ≤0.05 were considered as statistical significant.

## Results

### Increased fractalkine levels in diabetic individuals

**Fig. 1:**
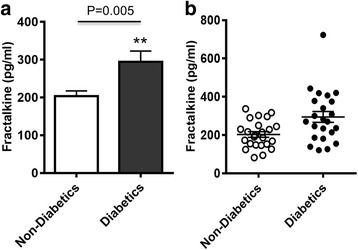
Plasma fractalkine levels in type-2 diabetic and non-diabetic individuals. Plasma fractalkine (CX3CL1) concentrations were measured in 23 type-2 diabetic (T2D) and 24 non-diabetic individuals using magnetic bead premixed 41-plex immune assays as described in Methods. The data expressed as (**a**) bar graph (mean ± SEM) and (**b**) cluster graph show that fractalkine levels were significantly higher in T2D (294 ± 28 pg/ml) as compared with no-diabetic (203 ± 15 pg/ml) subjects (*P* = 0.005)

Fractalkine has been shown to be involved in systemic inflammatory and metabolic disorders. However, the changes in the circulatory levels of fractalkine in T2D patients remain unclear. We, therefore, determined plasma fractalkine levels both in T2D and non-diabetic individuals. The data show that fractalkine levels in diabetic patients (294 ± 28 pg/ml) were significantly higher (P = 0.005) than in non-diabetic individuals (203 ± 15 pg/ml) (Fig. 1).

### Fractalkine in diabetic individuals associates with most of inflammatory chemokines

**Fig. 2:**
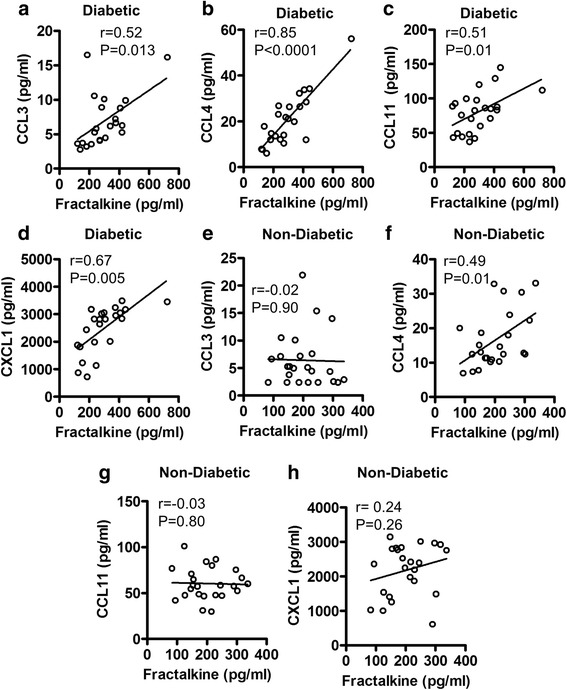
Association between plasma fractalkine and inflammatory chemokines in diabetic and non-diabetic subjects. Plasma levels of fractalkine and selective inflammatory chemokines were measured in 23 type-2 diabetic (T2D) and 24 non-diabetic individuals using magnetic bead premixed 41-plex immune assays as described in Methods. The data show that in T2D patients, a positive association was found between systemic fractalkine levels and those of (**a**) CCL3 (r = 0.52 *P* = 0.013); **b** CCL4 (r = 0.85 *P* < 0.0001); **c** CCL11 (r = 0.51 *P* = 0.01); and **d** CXCL1 (r = 0.67 *P* = 0.0005). However, in non-diabetic subjects, plasma fractalkine levels were found to associate only with (**f**) CCL4 (r = 0.49 *P* = 0.01); and not with (**e**) CCL3 (r = 0.02 *P* = 0.90); **g** CCL11 (r = −0.03 *P* = 0.80); and **h** CXCL1 (r = 0.24 *P* = 0.26)

Since the inflammatory chemokines are regarded as key mediators for inflammatory immune cell recruitment and induction of chronic sub-acute inflammation, we further asked if the changes in fractalkine expression were related with systemic inflammatory chemokines in T2D and/or non-diabetic subjects. To this end, our data show that the increased fractalkine levels in T2D patients (Fig. 2a–d) were positively correlated with systemic concentrations of CCL3 (r = 0.52, P = 0.013), CCL4 (r = 0.85, *P* < 0.0001), CCL11 (r = 0.51, *P* = 0.01), and CXCL1 (r = 0.67, *P* = 0.0005). In T2D individuals, fractalkine levels did not correlate with MCP-1 levels (r = −0.17, *P* = 0.42) (Additional file 1: Figure S1 While, in non-diabetic subjects (Fig. 2e–h), only the CCL4 was found to correlate positively with systemic fractalkine levels (r = 0.49, *P* = 0.01).

### Fractalkine in diabetic/non-diabetic individuals associates with inflammatory cytokines

**Fig. 3:**
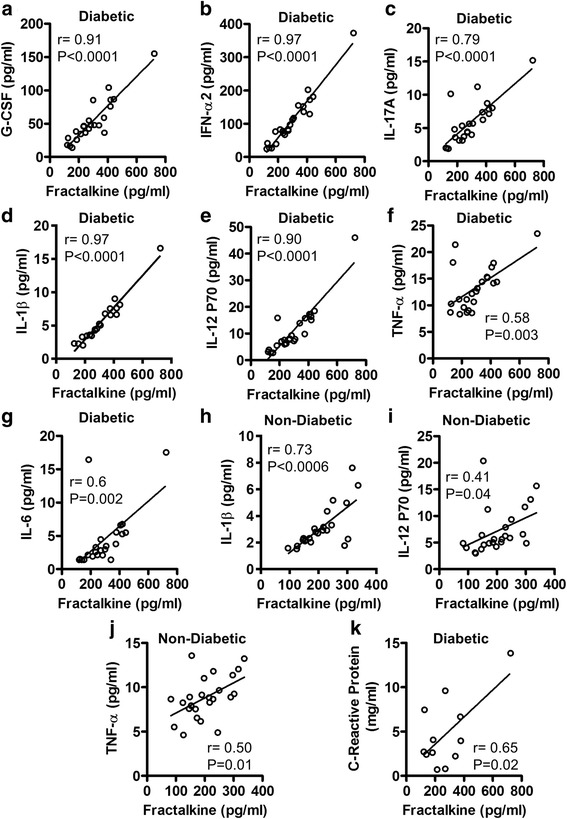
Relationship of plasma fractalkine levels with inflammatory cytokines in diabetic and non-diabetic subjects. Plasma levels of fractalkine and selective inflammatory cytokines were measured in 23 type-2 diabetic (T2D) and 24 non-diabetic individuals using magnetic bead premixed 41-plex immune assays as described in Methods. The data show that in T2D patients, plasma fractalkine levels correlated positively with those of (**a**) G-CSF (r = 0.91 *P* < 0.0001); **b** IFN-α2 (r = 0.97 *P* < 0.0001); **c** IL-17A (r = 0.79 *P* < 0.0001); **d** IL-1β (r = 0.97 *P* < 0.0001); **e** IL-12P70 (r = 0.90 *P* < 0.0001); **f** TNF-α (r = 0.58 *P* = 0.0003); and **g** IL-6 (r = 0.60 *P* = 0.002). While, in non-diabetic individuals, plasma fractalkine levels were found to associate with (**h**) IL-1β (r = 0.73 *P* < 0.0006); **i** IL-12P70 (r = 0.41 *P* = 0.04); and **j** TNF-α (r = −0.50 *P* = 0.01). **k** In T2D patients, systemic fractalkine levels also associated with circulatory C-reactive protein (CRP) levels (r = 0.65 *P* = 0.02); however, these data are shown for only 12 patients whose samples were still available for CRP assay

Cytokines are the key regulators of immune cell function and cytokine dysregulation or imbalance has been associated with various metabolic disorders. We, therefore, also wanted to know if the modulated fractalkine expression was associated with increased systemic levels of proinflammatory cytokines in these individuals. In this regard, we found that enhanced fractalkine levels in T2D patients were associated with G-CSF (r = 0.91, *P* < 0.0001), IFN-α2 (r = 0.97, *P* < 0.0001), IL-17A (r = 0.79, *P* < 0.0001), IL-1β (r = 0.97, *P* < 0.0001), IL-12P70 (r = 0.90, *P* < 0.0001), TNF-α (r = 0.58, *P* = 0.003), and IL-6 (r = 0.60, *P* = 0.002) levels in the circulation (Fig. 3a–g). In non-diabetic individuals, systemic fractalkine levels correlated positively with those of IL-1β (r = 0.73, *P* < 0.0006), IL-12P70 (r = 0.41, *P* = 0.04), and TNF-α (r = 0.50, *P* = 0.01) (Fig. 3h–j). Thus, as opposed to diabetics, the fractalkine changes in non-diabetic subjects did not associate with G-CSF (r = 0.31, *P* = 0.14), IFN-α2 (r = 0.23, *P* = 0.28), IL-17A (r = 0.10, *P* = 0.78), and IL-6 (r = 0.18, *P* = 0.45) levels (Additional file 2: Figure S2A-D). C-reactive protein (CRP) is a typical clinical marker of systemic inflammation and we next assessed whether the T2D-associated changes in fractalkine expression resonated with plasma CRP levels. To this end, we found that the elevated circulatory fractalkine levels in T2D patients correlated with plasma CRP levels (r = 0.65, *P* = 0.02) (Fig. 3k).

## Discussion

The increasing evidence supports that proinflammatory chemokines/cytokines are important players in establishing the conditions required for insulin resistance, T2D, and other related complications [2, 22–24]. As a chemoattractant and an adhesion factor, fractalkine orchestrates the inflammatory cell recruitment and colonization at sites of inflammation, such as expanding white adipose tissue in obesity and T2D. Fractalkine has been reported to be involved in monocyte adhesion to adipocytes and its expression by human adipocytes and stromal vascular cells was found to be associated with obesity, insulin resistance, and T2D [25]. In the present study, we report significantly higher plasma fractalkine levels in T2D patients as compared with non-diabetic individuals. In agreement with this finding, at least in part, Shah et al. also showed that plasma fractalkine levels were associated with adipose and metabolic traits and its levels were higher in T2D patients as compared with non-diabetic controls [25]. However, we did not find a significant difference of fractalkine levels between obese and control subjects which may be due to small numbers of subjects in each group. Also, it is not clear whether the obesity was a significant player in upregulating fractalkine expression in our diabetic patients but a human study of allergic asthma found that obesity was associated with enhanced fractalkine levels and leukocytic infiltration [26]. Although, the definitive cause(s) of higher fractalkine levels in patients with metabolic disease currently remain unclear, a link with obesity/T2D associated inflammation was reported [25, 27]. Both CX3CL1 mRNA and protein expression was found to be elevated in obese as compared with lean adipose tissue [25].

Our data further show that the elevated fractalkine expression in T2D patients correlates positively with the expression of other inflammatory chemokines such as CCL3, CCL4, CCL11 and CXCL1. The changes in plasma chemokines’ levels have been reported earlier in obesity and T2D [28]. CCL3 and CCL4 are the major chemotactic proteins produced by macrophages and adipose tissue. The positive association between fractalkine and these chemokines indicates that fractalkine, as a key regulator of leukocyte infiltration and adhesion, may be involved in the cross regulation of other chemokines. CCL11 or eotaxin-1 is predominantly an eosinophil chemotactic factor which is known to bind with CCR2, CCR3, and CCR5 chemokine receptors [29, 30]. CCL11, which is a chemoattractant for eosinophils, basophils, neutrophils, and monocytes, can play a significant role in the regulation of many inflammatory conditions including metabolic diseases. Notably, increased adipose tissue levels of multiple β-chemokines (CCL2, CCL3, CCL5, CCL7, CCL8, CCL11) and chemokine receptors (CCR1, CCR2, CCR3, CCR5) have been linked with obesity and associated metabolic inflammation [31]. CXCL1 or Gro-α which is expressed by macrophages, neutrophils, and epithelial cells, displays neutrophil chemoattractant activity and is a key regulator of hepatic neutrophil infiltration [32]. CXCL1 and CXCL5 were found to be elevated in the circulation with the onset of T2D and these chemokines were suggested to participate in the pathogenesis of T2D [9]. The patients with type-1 diabetes (T1D) or T2D were found to have elevated serum levels of CXCL1 as compared with non-diabetic controls [7, 8, 33].

We also found that fractalkine expression correlated positively with the circulatory levels of G-CSF, IFN-α2, IL-17A, and IL-6 only in diabetic subjects while it was found to be associated with IL-1β, IL-12p70, and TNF-α in both diabetic and non-diabetic individuals. These data suggest that fractalkine has a broader relationship with important proinflammatory cytokines and it may also have a differential association with these inflammatory proteins in the presence or absence of T2D. Our data indicate that the positive association of fractalkine with IL-1β, IL-12p70, and TNF-α may, therefore, be more consistent than its relationship with other proinflammatory cytokines including G-CSF, IFN-α2, IL-17A, and IL-6 as these cytokines were detected exclusively in T2D patients. TNF-α and IL-6 are considered the major players in chronic low-grade inflammation and insulin resistance. In partial agreement with our results, elevated levels of these proinflammatory cytokines were also reported in T2D patients by another study [34]. Chemokines, through activation of leukocytes, may lead to the induction of several proinflammatory cytokines including IL-1β, TNF-α, and IL-6. IL-17A is a proinflammatory cytokine which may be upregulated by fractalkine in T2D patients indirectly by the enhanced T cell infiltration and/or directly by the activation of Th17 cells in the adipose tissue. IL-17 stimulates the production of IL-6 in differentiated adipocytes and the enhanced IL-6 expression may in turn favor the proinflammatory Th17 subset differentiation [35]. In T2D patients, T cells are naturally skewed toward proinflammatory phenotypes (Th17 and Th1) that likely play a role to induce chronic sub-acute inflammation in T2D patients through the upregulated inflammatory cytokine production. In addition to IL-6, IL-1β and TGF-β can also induce Th17 differentiation in T2D patients [36, 37]. G-CSF is a cytokine and a hormone that acts as a colony stimulating factor for granulocytes and stem cells, as well as it also promotes the proliferation and differentiation of neutrophils. Our data showing the elevated levels of G-CSF in T2D patients may have significant implication as a recent study also indicated that G-CSF could be a major player in free fatty acid (FFA)-induced insulin resistance since the treatment with G-CSF led to insulin resistance in human adipocytes and myotubes [38]. Our data also show increased levels of IFN-α2 in T2D pateints which is a type-I interferon and it may be induced in these patients in the presence of danger signals from the dead or dying cells, such as during lipolysis observed in morbid obesity and T2D. Consistent with our data showing increased IL-12 levels in the peripheral circulation of both T2D and non-diabetic individuals, Suarez-Alvarez et al. also found elevated serum IL-12 levels in overweight and adult obese individuals and this IL-12 induction was found to strongly correlate with markers of low-grade inflammation and obesity [39]. IL-12 plays a role in the differentiation of naïve T cell into proinflammatory Th1 cells, it stimulates the production of IFN-γ and TNF-α, and also plays a critical role in the activation of T cells and NK cells [40]. We further found that in T2D patients, plasma fractalkine levels were associated with circulatory concentrations of CRP which is a signature clinical marker for systemic inflammation. These results are in agreement with other studies that also report the increased CRP levels in obese/T2D individuals [41, 42].

Fractalkine is the only member of CX3C chemokine family which is constitutively expressed by several hematopoietic and non-hematopoietic cells. The biological effects of fractalkine are mediated through its receptor CX3CR1 which is widely expressed on different cell types including monocytes/macrophages, NK cells, cytotoxic T cells, B cells, smooth muscle cells, tumor cells, microglia, and neurons. Given that, CX3CL1/CX3CR1 axis is found to play an important role in the pathophysiology of many inflammatory, infectious, neurological and neoplastic conditions. In regard with obesity/T2D, the emerging data support that CX3CL1/CX3CR1 axis promotes inflammation and, therefore, therapeutic targeting of this axis represents a promising translational approach which can be achieved, in principle, by: (1) controlling ligand (CX3CL1) shedding by using antagonists of ADAM-10/17 or cathepsin S; and/or (2) blocking cognate receptor (CX3CR1) by using specific antibodies or clinical grade CX3CR1 antagonists. These translational strategies hold promise for future studies targeting the CX3CL1/CX3CR1 axis to treat inflammation associated with metabolic diseases.

Overall, these correlative data support the fractalkine as a marker for metabolic inflammation in T2D patients; nonetheless, further studies involving larger multicenter cohorts will be required to validate these preliminary findings. It also remains to be seen whether fractalkine has a causal relationship with the elevation of these inflammatory proteins in T2D pateints and/or which inflammatory chemokines/cytokines can regulate the induction of fractalkine, individually or synergistically.

## Conclusions

Taken together, our data show that plasma fractalkine levels were significantly higher in T2D patients as compared with non-diabetic individuals. Fractalkine levels associated differentially with proinflammatory chemokines/cytokines as well as systemic CRP concentrations in diabetic and non-diabetic individuals which may have significant prognostic or therapeutic implications for T2D in humans.

## Additional files

Additional file 1: Figure S1. Plasma levels of fractalkine and selective inflammatory cytokines were measured in 23 type-2 diabetic (T2D) and 24 non-diabetic individuals using magnetic bead premixed 41-plex immune assays as described in Methods. The data show that in non-diabetic individuals, systemic fractalkine levels did not associate with (**A**) G-CSF (r = 0.31 *P* = 0.14); (**B**) IFN-α2 (r = 0.23 *P* = 0.28); (**C**) IL-17A (r = 0.10 *P* = 0.78); and (**D**) IL-6 (r = 0.18 *P* = 0.45). (TIF 716 kb)

Additional file 2: Figure S2. Plasma levels of fractalkine and selective inflammatory chemokines were measured in 23 type-2 diabetic (T2D) and 24 non-diabetic individuals using magnetic bead premixed 41-plex immune assays as described in Methods. The data show that in diabetic individuals, systemic fractalkine levels did not associate with systemic MCP-1 levels (r = −0.17 *P* = 0.42). (TIF 541 kb)

## Abbreviations

BMI: Body mass index
CRP: C-reactive protein
FBG: Fasting blood glucose
FFA: Free fatty acid
HbA1c: Glycated hemoglobin
MIP: Macrophage inflammatory protein
OGTT: Oral glucose tolerance test
T1D: Type-1 diabetes
T2D: Type-2 diabetes
