# Supplementary figures and images for: Increased circulatory levels of fractalkine (CX3CL1) are associated with inflammatory chemokines and cytokines in individuals with type-2 diabetes
Source: J Diabetes Metab Disord. 2017 Apr 4;16:15. doi: 10.1186/s40200-017-0297-3 (PMC5379731; doi:10.1186/s40200-017-0297-3)

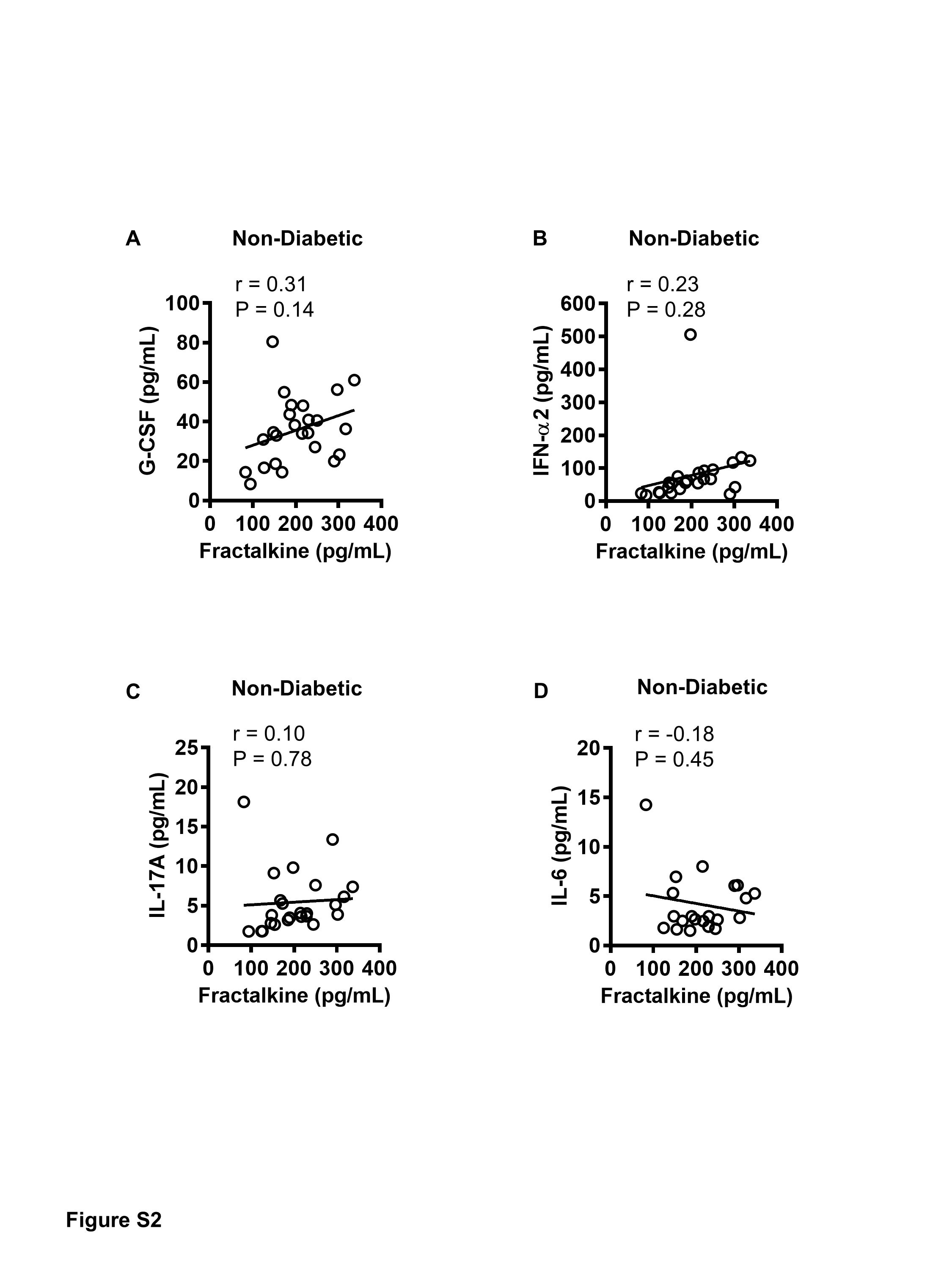

Supplement: Supplementary file 1 — Plasma levels of fractalkine and selective inflammatory cytokines were measured in 23 type-2 diabetic (T2D) and 24 non-diabetic individuals using magnetic bead premixed 41-plex immune assays as described in Methods. The data show that in non-diabetic individuals, systemic fractalkine levels did not associate with (A) G-CSF (r = 0.31 P = 0.14); (B) IFN-α2 (r = 0.23 P = 0.28); (C) IL-17A (r = 0.10 P = 0.78); and (D) IL-6 (r = 0.18 P = 0.45). (TIF 716 kb) [file 40200_2017_297_MOESM1_ESM.tif]

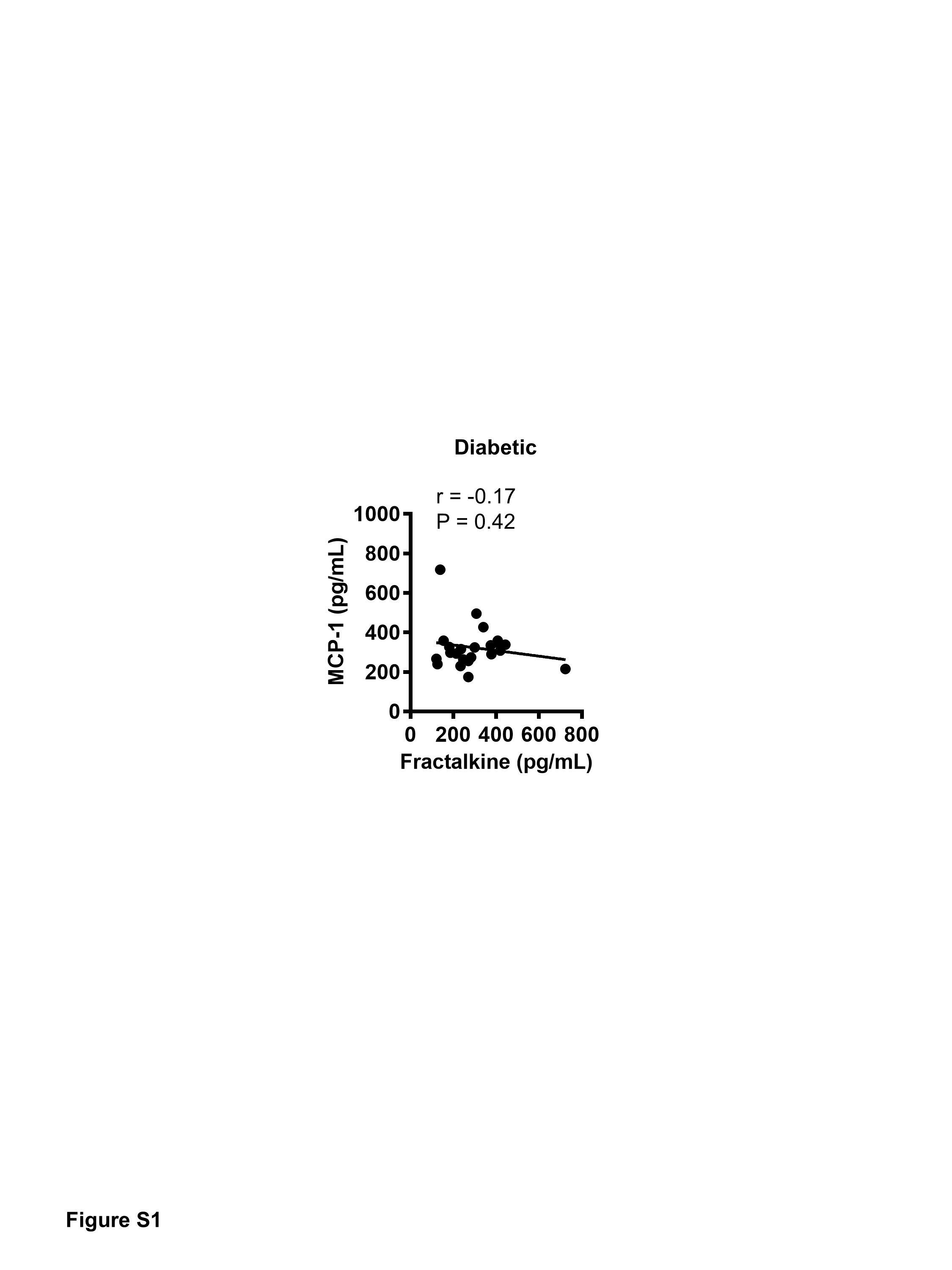

Supplement: Supplementary file 2 — Plasma levels of fractalkine and selective inflammatory chemokines were measured in 23 type-2 diabetic (T2D) and 24 non-diabetic individuals using magnetic bead premixed 41-plex immune assays as described in Methods. The data show that in diabetic individuals, systemic fractalkine levels did not associate with systemic MCP-1 levels (r = −0.17 P = 0.42). (TIF 541 kb) [file 40200_2017_297_MOESM2_ESM.tif]
